# Factors associated with health care provider knowledge on abortion care in Ethiopia, a further analysis on emergency obstetric and newborn care assessment 2016 data

**DOI:** 10.1186/s12913-019-4857-8

**Published:** 2019-12-30

**Authors:** Tefera Taddele, Theodros Getachew, Girum Taye, Misrak Getnet, Atkure Defar, Habtamu Teklie, Geremew Gonfa, Sheleme Humnessa, Aster Teshome, Zenebe Akale, Kasahun Mormu, Abebe Bekele

**Affiliations:** 1 Health System and Reproductive Health Research Directorate, Ethiopian Public Health Institute, 1242/1000 Addis Ababa, Ethiopia; 2 Department of Epidemiology and Biostatistics, Institute of Public Health, College of Medicine and Health Science, University of Gondar, Gondar, Ethiopia; 3 Maternal & Child Health directorate, Federal Ministry of Health, 43034/1000 Addis Ababa, Ethiopia; 4 The David and Lucile Packard Foundation, Addis Ababa, Ethiopia

**Keywords:** Comprehensive abortion care, Providers’ knowledge, EmONC, Knowledge score

## Abstract

**Background:**

Abortion is one of the major direct causes of maternal death, accounting for 7.9% globally. In Africa, 5.5 million women have unsafe abortions annually. Although maternal deaths due to complications of abortion have declined in Ethiopia, women still die from complications. Few studies have focused on providers’ clinical knowledge. This study investigates the level of health workers’ knowledge of comprehensive abortion care and its determinants in Ethiopia.

**Methods:**

Data from the national emergency obstetric and newborn care (EmONC) assessment was used. A total of 3804 facilities that provided institutional deliveries in the 12 months before the assessment were included. Provider knowledge was assessed by interviewing a single provider from each facility. Criteria for selection included: having attended the largest number of deliveries in the last one or two months. A summary knowledge score was generated based on the responses to three knowledge questions related to immediate complications of unsafe abortion, how a woman should be clinically managed and what the counselling content should contain. The score was classified into two categories (< 50% and > =50%). Logistic regression was used to determine individual and facility-level factors associated with the summary knowledge score.

**Result:**

A total of 3800 providers participated and the majority were midwives, nurses and health officers. On average, providers identified approximately half or fewer of the expected responses. The multivariate model showed that midwives and nurses (compared to health officers), being female, and absence of training or practice of manual vacuum aspiration were associated with lower knowledge levels. Important facility level factors protective against low knowledge levels included employment in Addis Ababa, being male and having internet access in the facility.

**Conclusion:**

To increase knowledge levels among providers, pre- and in-service training efforts should be particularly sensitive to female providers who scored lower, ensure that more midlevel providers are capable of performing manual vacuum aspiration as well as provide special attention to providers in the Gambella.

## Background

The World Health Organization (WHO) defines unsafe abortion as “a procedure for terminating an unintended pregnancy, carried out either by persons lacking the necessary skills or in an environment that does not conform to minimal medical standards, or both” [1]. It is estimated that of the 210 million pregnancies that occur each year, 80 million are unintended, which is a root cause of induced abortion [2, 3]. Abortion is one of the direct causes of maternal deaths that accounts for 7.9% of global maternal deaths [4]. Of the 42 million pregnancies that end in induced abortion each year, 20 million are unsafe [5].

In Ethiopia, unsafe abortion estimated 32% of maternal deaths before 2005 [6]. Maternal mortality in Ethiopia due to unsafe abortion has declined during the past decades because of the introduction of safe and effective technologies such as manual vacuum aspiration (MVA), medical abortion, training on comprehensive abortion care (CAC) for mid-level and higher health care providers and the provision of legal safe abortion service [6].

The pregnancy termination reform represented a step forward to reducing maternal mortality in Ethiopia and has resulted in the expansion of comprehensive abortion care [7]. Before the reform of the country’s abortion law, abortion was allowed in Ethiopia only if two physicians, including at least one gynaecologist, agreed that pregnancy termination would avert “grave or imminent danger” to the woman [8]. The revised law, enacted by Ethiopia’s parliament in 2005, allows a woman to obtain a safe and legal abortion if: pregnancy is due to rape or incest; she a has physical or mental disability, she is physically or mentally unprepared for childbirth; the pregnancy would put her life or physical health at risk if she continues her pregnancy, or is younger than 18 and physically health at risk [9].

Comprehensive abortion care is critically important in countries like Ethiopia where the estimated number of pregnancies that end in abortion is about half a million annually [10]. However, according to government sources, only 181, 812 clients received safe abortion care in 2013/14 with a slight improvement from the preceding year’s performance of 138,303 cases [10]. Meeting the comprehensive abortion care needs of the population has been limited; fewer than one-quarter of the recommended number of facilities can provide the essential services [8]. Many east African women including Ethiopia are restricted from accessing abortion services due to a shortage of health providers trained in comprehensive abortion care [11].

Historically, abortion-related studies conducted in Ethiopia have focused mainly on the met need for family planning [12, 13], reasons for abortion [14], the estimation of how many are performed [15, 16], the distribution of abortion services [17] and patient satisfaction [18]. While several studies have examined comprehensive abortion care, they tend to be small scale studies. There is limited information about the level of health workers’ clinical knowledge on comprehensive abortion care and factors associated with that knowledge. This study aims to fill that gap.

## Methods

### Study design

Data were used from the national 2016 emergency obstetric and neonatal care assessment. Detailed methods were presented in the assessment report [19]. Briefly, data were collected from May to October, 2016 at all facilities in all 9 regions and 2 city administrations in the country that had provided care for institutional deliveries in the 12 months preceding the assessment.

The type of facilities included in this study were hospitals (referral, general and primary), health centres, Maternal Child Health (MCH) speciality centres, MCH speciality clinics and higher clinics. Rural and urban facilities were also included. From 3804 health facilities assessed, a total of 3800 health care providers were included in the analysis. Provider knowledge on abortion care was assessed by interviewing one provider from each facility. Selection criteria for the provider were: 1) the health worker who attended the largest number of deliveries in the last month or if no births had been reported in the facility in the previous 30 days, in the last two months,, and 2) was physically present when data collectors visited the facility. If the selected provider refused to provide consent, he or she was not replaced by another at that facility.

### Data analysis

The dummy variables entered in the regression were sex, qualification, MVA training and MVA service provision of health care providers were collected, the availability of internet, computers, safe abortion care, and family planning guidelines. Health care providers were asked a series of questions related to unsafe abortion: “What are the immediate complications of unsafe abortion?”; “What do you do for a woman with an unsafe or incomplete abortion?”; and, “What information do you give to clients after unsafe or incomplete abortion?” A summary knowledge score was generated based on these questions. Each knowledge question had multiple possible “correct” answers; that is, answers that respondents were expected to provide spontaneously. Respondents were scored on each question by calculating the number of correct responses provided out of the total possible, and standardizing this to a scale of 100. A one way ANOVA statistical test was used to compare the level of knowledge among different cadre of health care providers. The outcome variable was overall knowledge score, which was divided into two categories (> = 50 and < 50%). A score higher than 50% was considered acceptable [20]. The Medical Council of India recommends 50% as the minimum pass mark for all summative examinations in medical specialties. The National Board of Examination in India also accepts overall 50% marks as a minimum acceptable mark for passing in Objective Structure Clinical Examination (OSCE) [21]  and a score higher than 50% was also considered acceptable [22]. Accordingly, we operationally defined those scores 50% and above as a passing score. For the logistic regression, the overall abortion care knowledge score was based on the sum of all three questions and their 22 possible responses, and classified into two categories (< 50% labelled as 1, and > =50% labelled as 0). The dependent variable of interest was those providers who scored below 50%. We used in bivariate analysis a wider confidence interval (80% CI) in order not to miss potential factors that might affect provider low knowledge. Our main interest in the dependent variable category was those providers who scored below 50. Finally, a multivariable logistic regression model was used to determine independent individual and facility-level factors associated with the knowledge score at a 5% significance level. The analysis was done using STATA Version 14.

### Ethical issues

Ethical clearance was obtained from the Ethiopian Public Health Institute (EPHI) scientific and ethical review board. Informed verbal consent was taken from all facilities and all selected health workers. No incentive was provided for participation. No personal identifier was used to maintain confidentiality and data were maintained on a password protected EPHI server.

## Result

### Demographic characteristics and providers’ experiences

Only four respondents refused the interview, which resulted in a response rate of 99.9%. Most respondents were midwives with either diploma or BSc (84%) followed by nurses (12%) with a diploma or BSc degree. Most respondents (96%) were from public/government-owned facilities. The majority of providers were from health centres (91%), followed by primary hospitals (4%). More than six out of ten respondents were female (63%). The average respondent’s age was about 26 years. The mean number of deliveries attended in the month before the visit to the facility was about 19. Among providers with at least three years of experience, the mean number of facilities that providers had worked was nearly 2 (see Table 1).

**Table 1:** Health care providers’ demographics characteristics and professional experiences (*n* = 3800)

National	Providers interviewed	
	3800	100
Health worker cadre		
MD (general practitioner)	4	0
Midwife (BSc or diploma)	3193	84
Nurse (BSc or diploma)	456	12
Health officer	143	4
Other^a^	4	0
Facility type		
Referral/specialized hospitals	30	1
General hospitals	103	3
Primary hospitals	160	4
MCH specialty centers	23	1
Health centers	3455	91
MCH specialty clinics	16	0
Higher clinics	13	0
Managing authority		
Public/government	3658	96
Private-for-profit	83	2
Private-not-for-profit^b^	59	2
Characteristics		
Sex		
Female	2409	63
Male	1391	37
Mean age (in years)	25.7	
Professional experience		
Mean number of deliveries attended in past month	19.3	
Mean number of years at current facility	2.0	
Mean number of years since receiving professional qualification	3.2	
Number of providers who have had qualification for ≥3 years	1634	
Among them, mean number of different health facilities posted to in past 3 years	1.7	

^a^Other health worker cadres include emergency surgical officers and health extension practitioners

^b^Includes NGO, faith-based, or mission facilities

### Health providers’ knowledge scores on abortion

Health care providers were asked a series of questions related to unsafe abortion.

On average, providers could name 51% of the five immediate complications of unsafe or incomplete abortion (bleeding, sepsis, shock, genital injuries and abdominal injuries). On average, health officers, midwives and nurses were able to identify 60, 51 and 47% of the complications, respectively. No statistically significant differences were observed among the interviewed health cadres on knowledge of complication of unsafe abortion (*p*-value = 0.735). The most frequently identified complication was bleeding (85%) and the least was abdominal injuries (17%).

Based on the 10 possible responses to the question about what to do for a woman with complications of unsafe or incomplete abortion, the summary average score was 46%. Begin IV fluids were the item mentioned most frequently by all health provider groups with an overall score of 82%, followed by beginning antibiotics (73%). Manual vacuum aspiration was mentioned by 51% of the providers, while the least mentioned item was performing evacuation with curettage (13%). The overall score of providing ergometrine, oxytocin or misoprostol to treat hemorrhage was mentioned by 26%. Average knowledge scores were also not significant for what to do for a woman with complications of unsafe or incomplete abortion among interviewed health care providers (*p*-value = 0.417).

The average summary knowledge score concerning giving information to women experiencing unsafe or incomplete abortions was 41%. On average health officers mentioned 46% of the seven possible responses, followed by midwives (42%) and nurses (37%). The most frequently identified information was counselling on family planning and services and the least mentioned was refering for precancerous cervical lesion screening. Again, at the 5% level of significance, we saw no differences among cadres’ average knowledge scores (*p*-value = 0.799). Nevertheless, it is worth noting that nurses scored lower than other cadres across all questions (See Table 2).

**Table 2:** Providers’ knowledge scores on complications of unsafe abortion, interventions and counselling content, by health cadre

	Total	Midwives	Nurses	Health officers	*P* value
	*n* = 3792	*n* = 3193	*n* = 456	*n* = 143	
What are the immediate complications of unsafe abortion?					
Average knowledge score (out of 100)	51	51	47	60	0.735
Percent providing specific response:					
Bleeding	85	85	82	92	
Sepsis	72	72	63	83	
Shock	51	51	47	63	
Genital injuries	29	29	28	40	
Abdominal injuries	17	17	13	24	
When you see a woman with complications from an unsafe or incomplete abortion, what do you do?					
Average knowledge score (out of 100)	46	46	40	52	0.417
Percent providing specific response:					
Begin IV fluids	82	82	77	88	
Begin antibiotics	73	74	64	85	
Perform (manual or electric) vacuum aspiration	51	53	31	49	
Assess vital signs	50	51	39	59	
Assess vaginal bleeding	46	47	43	56	
Refer	46	46	54	44	
Do a vaginal exam	39	39	36	49	
Provide counselling	30	31	21	41	
Give Ergometrine or oxytocin or misoprostol	26	26	21	29	
Perform evacuation with curettage	13	13	10	22	
What information do you give clients who were treated for an unsafe or incomplete abortion?					
Average knowledge score (out of 100)	41	42	37	46	0.779
Percent providing specific response:					
Counselling on family planning and services	85	87	71	87	
Refer for family planning to receive family planning methods	53	55	38	59	
Inform when a woman can plan to conceive again	51	52	42	53	
Describe consequences of an unsafe abortion	47	46	48	56	
How to prevent reproductive tract infections/HIV	30	29	31	38	
Social support	17	17	18	19	
Refer for precancerous cervical lesion screening	7	6	11	12	

### Factors associated with providers’ knowledge on comprehensive abortion care

The knowledge score was classified into two categories, those who responded below 50% and those who scored 50% or higher. At 80% CI, the variables with a significant association with provider’s to have low knowledge were: provider’s professional qualification, sex, whether he or she had performed MVA, had been trained to perform MVA, type of facility, location of facility, availability of computer, internet, and the availability of safe abortion care and family planning guidelines in the workplace.

Among the provider level factors, providers’ profession was found to be a statistically significant factor for predicting knowledge. So, the result showed that midwives are almost twice and nurse 3 times as likely as their health officer colleagues to have a low knowledge score. Compared to females, males are less likely (OR .78) to have a low knowledge score.

Similarly, training to perform MVA and actual provision of MVA in the last three months significantly protected against a lower summary score.

Among the facility level factors in the model, region and availability of internet in the workplace were significantly associated with providers’ knowledge score. Providers in Somali and Benishangul-Gumuz regions and Addis Ababa city administration were less likely to have a low-level knowledge score (< 50%) when compared to providers in the Tigray region. On the other hand, providers working in the Gambella region were 8 times more likely to have a low summary knowledge score than providers in the Tigray. Providers with access to the internet were less likely by almost 30% to have a low-level knowledge score (See Table 3).

**Table 3:** Factors associated with health care providers’ knowledge on abortion care

Variables	Unadjusted		Adjusted	
	Odds ratio	*P*-value	Odds ratio	*P*-value
Provider level variables				
Provider qualifications				
Health Officer	1		1	
Midwife	1.84	< 0.00011	1.96	< 0.0001
Nurse	3.17	< 0.0001	2.99	< 0.0001
N. of facilities posted previously in the last 3 years	1.00	0.399		
Experience in current facility (number of years)	0.99	0.354		
Provider’s age	0.99	0.354		
Provider’s sex				
Female	1		1	
Male	0.78	< 0.0001	0.77	0.001
Performed manual vacuum aspiration (MVA) in the last 3 months				
No	1		1	
Yes	0.50	< 0.0001	0.67	< 0.0001
Trained to perform MVA				
No	1		1	
Yes	0.65	< 0.0001	0.74	< 0.0001
Facility level variables				
Region				
Tigray	1		1	
Afar	1.12	0.669	1.02	0.947
Amhara	0.89	0.425	0.79	0.129
Oromia	1.28	0.074	1.05	0.739
Somali	0.83	0.344	0.58	0.013
Benishangul Gumuz	0.52	0.052	0.47	0.029
SNNP	1.13	0.414	0.83	0.239
Gambella	6.37	0.003	7.95	0.006
Harari	1.19	0.744	1.34	0.597
Addis Ababa	0.59	0.01	0.58	0.018
Dire Dawa	0.88	0.77	0.79	0.613
Managing authority				
Public	1		1	
Private for profit	0.76	0.215	1.35	0.257
Private for non-profit	0.82	0.452	0.90	0.705
Types of facility				
Hospital/MCH specialty centre	1		1	
Health centre/clinics	1.89	< 0.0001	1.26	0.13
Facility location				
Urban	1		1	
Rural	1.47	< 0.0001	1.15	0.084
Availability of computer				
No	1		1	
Yes	0.74	< 0.0001	0.90	0.221
Availability of internet				
No	1		1	
Yes	0.44	< 0.0001	0.69	0.023
Availability of safe abortion care guideline				
No	1		1	
Yes	0.66	< 0.0001	0.88	0.133
Availability family planning guideline				
No	1		1	
Yes	0.82	0.012	0.94	0.507

## Discussion

The present study has provided comprehensive evidence on the knowledge of health care providers on CAC at the national level. Overall, providers on average were able to identify 51% of immediate complications of unsafe or incomplete abortion, 46% of what to do for a woman with complications and 41% of the items related to the information to be given to women with abortion complications. Although differences in the summary scores for each separate knowledge question across the professional categories were not significant, once they were combined for the dependent variable of the regression analysis, professional qualification was a predictor of knowledge. There was a significant difference in the overall score according to the logistic regression model that showed midwives and nurses with lower knowledge scores compared to health officers. Other individual-level predictors were the sex of the provider, training in MVA and recent practice of MVA. Facility factors that also significantly predict knowledge were region and availability of the internet.

The technical and procedural guideline for safe abortion that was prepared by the Ethiopian ministry of health in 2014 state that in order for health care providers to effectively carry out their responsibilities, they should acquire basic knowledge and skills during pre-service training and receive periodic updates through on-the-job training [6].

In this study, professional categories were a factor in predicting overall knowledge of abortion. Elsewhere professional category and PAC training have also been found to be significantly associated with the knowledge and skills of providers on abortion [22]. The most identified immediate complication of unsafe abortion was bleeding. Similarly, providers in Afghanistan also identified bleeding as the most recognized complication of unsafe or incomplete abortion but at a lower proportion (66%) compared to our finding [22].

The present study has several strengths. First, the study covered all administrative regions in Ethiopia and the census of facilities that provided delivery services in the past 12 months before the study. Also, the analysis focused on critical aspects of abortion care providers that have not been covered by most studies. The response rate was 99.9% which may reflect the knowledge of all respondents. On the other hand, it has some limitations. This study assessed knowledge based on only 3 open-ended questions. Other types of questions-such as pointed questions about technical issues, true-false questions, multiple-choice questions, scenarios or simulations would all have provided a richer assessment of provider knowledge. This study did not assess perceptions, beliefs, behaviours or other socially related factors that influence providers and affect the quality of care. These factors could show programmers, policymakers, and health care managers where further interventions might be needed. It was also beyond the scope of this study to interview clients to better understand their experience and interactions with providers that would provide further evidence about abortion care.

## Conclusion

An accurate initial physical assessment and main treatment by knowledgeable health professional is essential to ensure appropriate treatment and prompt referral for complications of unsafe abortion.

Our findings showed that overall knowledge was low, based on a composite index that measured knowledge about abortion care.

Based on these findings, we recommend the provision of pre-service and refresher training with appropriate practical engagement for all providers on the most immediate complications of unsafe or incomplete abortion, clinical management and counselling content that meet national and international standards. We suggest emphasis be given to nurses and midwives as they scored lower than health officers and they are key frontline providers at health centres.

Health facilities should be well equipped with infrastructure and opportunities that promote learning and increase knowledge such as the internet. Finally, efforts to improve equity in training of all kinds may minimize regional disparities.

Survey data cannot tell the full story of any situation and so we strongly advocate further studies with different methodologies to improve our understanding of health care providers’ perspectives on the topic of abortion.

## Data Availability

The datasets used during the current study are available from the corresponding author on reasonable request and based on EPHI’s data sharing guideline. If needed, we can share the dataset specifically used for this study to the journal.

## Abbreviations

AMDD: Averting Maternal Deaths & Disability
BEmONC: Basic Emergency Obstetric & Newborn Care
BSc: Bachelor of Science
CAC: Comprehensive abortion care
EmONC: Emergency obstetric & newborn care
EmWA: Ethiopian midwifery Association
EPHI: Ethiopian Public Health Institute
EtD: Evidence to Decision
IV: Intravenous
JSI: John Snow International
MCH: Maternal & child Health
MVA: Manual Vacuum Aspiration
OSCE: Objective Structure Clinical Examination
PAC: Post abortion Care
UNFPA: United Nations Population Fund
UNICEF: United Nations Children’s Emergency Fund
WHO: World Health organization
